# Bibliology

**Published:** 1908-10-15

**Authors:** 


### BIBLIOGRAPHY.

Black's Operative Dentistry.—At last the great work on Operative Dentistry by Prof. G. V. Black, is published. It is a two volume work of over 700 pages in two volumes; with over 600 illustrations, all new and well executed, and printed on fine paper, many of them are bound into the work as inserts. This enables the author to utilize many of his beautiful microscopic preparations in illustration of his text. It has been impracticable for us to read the entire work for a critical review, as every subject is written in so new and instructive a style that one can hardly give it a casual reading. The first volume is given to the consideration of the pathology of the hard tissues and its treatment. The first chapter is of forty pages, devoted to atrophy of the teeth. Each phase of atrophy is scientifically studied as to its etiology and practical treatment. We know of no other place where this subject gets as complete consideration. The author's treatment is practicable, but may not pass without some further suggestions in respect to the selection of filling materials that are available. He also takes up erosion, and shows that this disease is more common than the average dentist thinks. He gives illustrations of the various forms of erosions, and makes the statement that some practitoners seldom see a case of erosion; while he cites one dentist who sent him over fifty casts of erosions, in all degrees of wasting of the tooth structure. The author cites various attempts to discover the cause of erosion, but finds some flaw in each of them, and concludes that the true cause of erosion is at present unknown. He also concludes that no present method can be supported as a specific treatment. He is inclined to rely on the technical rather than the medicinal. The author then takes up the subject of dental caries, and in nearly a hundred pages he gives a complete historical statement of what we now know of the etiology of dental caries and its treatment. As all are familiar with Dr. Black's work on this subject it will not be necessary for us to rehearse it here. He gives an excellent description of the history and character of the researches made on this subject and the results obtained.

The next 125 pages are devoted to the more practical techical matters, such as the "management of patients," "force used in mastication," "sensitiveness of the dentine," etc., light and care of the eyes. The treatment of dental caries by prophylaxis and filling of cavities, the selection of proper filling materials for their therapeutic and physical properties make up another chapter.

The next chapter considers the principles involved in the management of cavities by classes, under five different classes, historically and technically considered. First, pits and fissures; 2nd, bicuspid and molar proximal cavities; 3rd, simple proximal incisor cavities; 4th, compound proximal cavities; 5th, labial and buccal cavities; and then special cavities.

The first volume closes with a chapter on the management of children's teeth. Development and shedding of the deciduous teeth and their treatment.

This volume contains twenty-five pages of definitions of technical terms involved in operative literature arranged alphabetically, which, in itself, is one of the very valuable features of the book.

The second volume is given over entirely to a consideration of the technical details of operative dentistry. First, the operative nomenclature, as pertaining to the teeth and cavities; then instruments, their nomenclature, classification and methods of using to the best advantage, including the rubber dam and dental engine and their uses. Cavity preparation is very elaborately considered with reference to technical and therapeutic treatment in more than a hundred pages with full illustrations of instruments and cavity forms.

The physical properties of filling materials is also very elaborately treated, including gold, amalgam, porcelain, cement and gutta-percha.

Naturally this part of the book receives at Dr. Black's hands careful scientific treatment, as well as his mature judgment of materials and the best methods for using them. Those who are familiar with Dr. Black's writings for the past twenty-five years will find a rare treat in going over this book, in which they will find a re-statement of his long scientific research and practical handling of the subject of operative dentistry. It is a "Black" book from beginning to end. What is more it is not a rehash of operative dentistry, crown and bridge work, orthodontia, and everything else in practical dentistry. It is not made up of contributions by a dozen or twenty authors, but is a one man book, and is therefore consecutive and full of character. There is little repetition, although a large work it is not unnecessarily elaborated. In fact, the only criticism we are tempted to make is that the author has not given the subject of gold and porcelain inlays the same careful scientific scrutiny he has the metallic and plastic operations, and the cement filling while having a reasonably clear and precise presentation is not discussed in the careful detail it deserves. It is a grand work, however, and any one who fails to possess and carefully read it will be doing himself a great injustice. It is the life work of one of the greatest dental scientists who ever lived and a man who has had an unusual opportunity for developing a text book as well as a treatise on the most important phase of our profession. It is a standard and classic work that will endure, and the profession is greatly benefited as well as honored in this grand work. It is published by the Medico-Dental Publishing Co., of Chicago, at ten dollars ($10.00) for the two volumes.

